# Temporal Limitations of the Standard Leaky Integrate and Fire Model

**DOI:** 10.3390/brainsci10010016

**Published:** 2019-12-27

**Authors:** Liya Merzon, Tatiana Malevich, Georgiy Zhulikov, Sofia Krasovskaya, W. Joseph MacInnes

**Affiliations:** 1 Vision Modelling Laboratory, National Research University Higher School of Economics, 109074 Moscow, Russia; geole@mail.ru (G.Z.); krasov.sofia@gmail.com (S.K.);; 2 Department of Psychology, National Research University Higher School of Economics, 101000 Moscow, Russia; 3 Neuroscience and Biomedical Engineering Department, Aalto University, 02150 Espoo, Finland; 4 Werner Reichardt Centre for Integrative Neuroscience, 72076 Tuebingen, Germany; t.v.malevich@gmail.com; 5 Institute of Water Problems Russian Academy of Science, 117971 Moscow, Russia

**Keywords:** saccade generation, salience model, visual search, leaky integrate and fire model

## Abstract

Itti and Koch’s Saliency Model has been used extensively to simulate fixation selection in a variety of tasks from visual search to simple reaction times. Although the Saliency Model has been tested for its spatial prediction of fixations in visual salience, it has not been well tested for their temporal accuracy. Visual tasks, like search, invariably result in a positively skewed distribution of saccadic reaction times over large numbers of samples, yet we show that the leaky integrate and fire (LIF) neuronal model included in the classic implementation of the model tends to produce a distribution shifted to shorter fixations (in comparison with human data). Further, while parameter optimization using a genetic algorithm and Nelder–Mead method does improve the fit of the resulting distribution, it is still unable to match temporal distributions of human responses in a visual task. Analysis of times for individual images reveal that the LIF algorithm produces initial fixation durations that are fixed instead of a sample from a distribution (as in the human case). Only by aggregating responses over many input images do they result in a distribution, although the form of this distribution still depends on the input images used to create it and not on internal model variability.

## 1. Introduction

Despite limits to the processing capacity of the human visual system, we are quick to make sensible interpretations of incoming visual information. This ability to select information from our complex environment is commonly ascribed to attention, the focus of which could be likened to a spotlight moving across the visual field that highlights its most relevant areas [1]. Shifts of attention can be endogenous, i.e., top-down and goal-directed, or exogenous, as in bottom-up and driven by external factors such as perceptual properties of visual stimuli [1,2].

Dominant models of bottom-up attention in tasks like inspection and visual search rely on the idea that visual saliency influences where we attend; in other words, they assume that properties of a visual stimulus stand out against properties of other environmental stimuli and capture our attention [3,4,5,6]. This concept is based on the feature integration theory (FIT) of attention [7], which states that, at an early ‘pre-attentive’ processing stage, features are registered in parallel across the whole visual field and encoded along a number of perceptual dimensions (orientation, color, spatial frequency, brightness, etc.), and, at a later ‘attentive’ stage, they are combined to a perceived object with help of attention [4,7].

This concept could be implemented on a level of computational model. In Itti and Koch’s model, low-level features (for the classical implementation, they are orientation, colors, and intensity) are extracted from the input image and represented as separate feature maps. At the next step, which reflects an ‘attentive’ stage, these feature maps are normalized and integrated across spatial coordinates into a higher representation, i.e., a saliency map, and the location of the most salient stimulus ‘wins’ a competition between neurons and is therefore attended [6,8]. The final layer of these models of fixation selection are typically implemented with a winner-take-all (WTA) network of neurons using a leaky integrate and fire (LIF) model, which is a neuronal activation model able to predict neuronal spikes [8]. To prevent refixations at the most salient locations, the model implements inhibition of return (IOR), i.e., a mechanism that is commonly believed to impede returning to the recently attended locations and promotes novelty in visual search [9,10] (see also [11] for an alternative point of view on IOR).

The Saliency Model is thought to be biologically accurate with center–surround receptive field interactions in visual pathways implemented with a pyramidal architecture of the model; feature-specific sensitivity of neurons in early visual cortex of the brain, and neuronal activation as spiking patterns simulated in the LIF component of the WTA layer [6,12]. Also, this family of models has been shown to produce a reasonable fit to human data in terms of spatial localization of salient stimuli [4,6,13]. Overt attention, in accordance with the definition by Posner [2], is operationalized as the distribution of gaze fixations across an image (see also the MIT saliency benchmark [14] for accuracy characteristics of spatial predictions produced by different implementations of the Saliency Model against human data).

The early parallel feature maps of the model combine to produce a saliency map that provides location and intensity information, and this is the first step in generating the spatial predictions of fixation locations. The LIF is a two-dimensional neural network model that simulates neuronal spikes, with each pixel of the saliency map being treated as single neuron representing a neuronal population with very strong synaptic connections [4]. Artificial neurons in the Saliency Model are implemented by using a differential equation to simulate the build-up of charge potential at a location and to fire a pulse once a threshold has been reached. The model for artificial neurons is called leaky integrate-and-fire, and, despite its relative simplicity, it was shown to predict single cell firing patterns with high accuracy [15,16]. The predictions for temporal distribution of these fixations are generated by the LIF neurons in the WTA layer based on the one-time input of the salience map.

After nearly 20 years, the standard salience model [4,5] is still used as a solid implementation of our theoretical understanding. It has inspired many further modifications of the model, including various attempts to add different implementations of a top-down attentional component [6,8] (for review, see [17]). However, other algorithms have surpassed the Saliency Model in fixation prediction and classification. Models based on deep convolutional networks, in particular, have shown better performance than other models in terms of spatial prediction accuracy. Indeed, according to the MIT Saliency Benchmark [14], the original Saliency Model, proposed by Itti and Koch [4,18], has moderate accuracy: the AUC-Judd metric, a version of the Area Under ROC curve [19], is equal to 0.6, while the best result in the Benchmark is reached by a model with a deep network whose accuracy is 0.88 (or 0.84 for the same model without including center bias in the model). Nonetheless, the model has its advantage as interpretable and theoretically grounded.

The original salience model is one of a very few models that can not only predict spatial distribution of attention, but also its temporal dynamics. Moreover, other models that include a temporal component only predict the scan path (order of the fixations) and not the time course of fixations latency. One key advantage of the classic model over deep-learning spatial models has been its ability to generate humanlike fixation times, as well as spatial predictions. This ability of the Itti and Koch’s Saliency Model [4] to provide temporal predictions of overt attention is the focus of the current research. The latency of saccades in a viewing or a search tasks is typically measured as the time duration of the intervening fixation and the temporal profile of these fixations in search tends to have a distinct positively skewed distribution [20,21,22]. While the LIF model has been tested for its neural spiking accuracy, however, there is little research on how well the classic model itself reproduces temporal dynamics of overt attention. The past few decades have brought a number of other approaches addressing temporal accuracy of responses [23,24,25] that simulate a typical profile of the temporal data well. However, these approaches address the temporal aspect only, with the location of attentional shift in abstract space. The Saliency Model remains one of the few that, in principle, has the capability to simulate both aspects with relation to the underlying biological mechanisms. In the present study, we test the temporal accuracy of the classic LIF + WTA combination and determine which parameter space of the Saliency Model accurately fits temporal aspects of human data, if any.

**Proposal:** Specifically, given the Itti and Koch model’s [4] longevity and its ability to generate saccades using LIF, we wanted to test the model’s accuracy in reproducing latency distributions against human data from a visual search task. Multiple attempts of optimal parameter choice were used to match human data, with the initial attempt being the default parameter settings found in Walther and Koch’s [5,26] implementation. Following this step, we trained the LIF by adjusting its default parameters with a genetic algorithm (GA) [27] and the MATLAB built-in optimizer *fminsearch*, which uses the Nelder–Mead (NM) method [28,29] to find the optimal parameter space for temporal predictions. While these LIF parameters are also involved in spatial prediction (via the WTA layer), our optimization only considered the temporal accuracy for this initial stage. Therefore, if the LIF is able to simulate an accurate temporal distribution, it would be reasonable to search for a parameter space where spatial and temporal accuracy would both be high.

## 2. Materials and Methods

### 2.1. Data Collection

**Figure 1:**
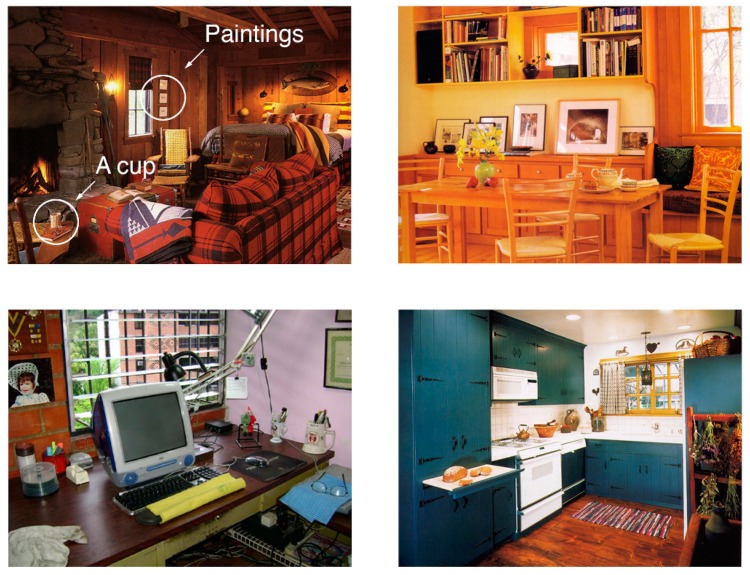
The examples of visual stimuli taken from the LabelMe database [31].

The model’s predictions were tested against human data collected from 91 test images presented on a 21″ LCD monitor as a part of visual search experiment. Human data were collected as a part of a previous study [30]. Participants (*N* = 18) performed a visual search task on the images of natural indoor scenes taken from the LabelMe open database [31] (see the examples in Figure 1). The total amount of obtained data contained 35 blocks of trials for a visual task (18 participants × 2 blocks, one block was excluded as incomplete).

**Figure 2:**
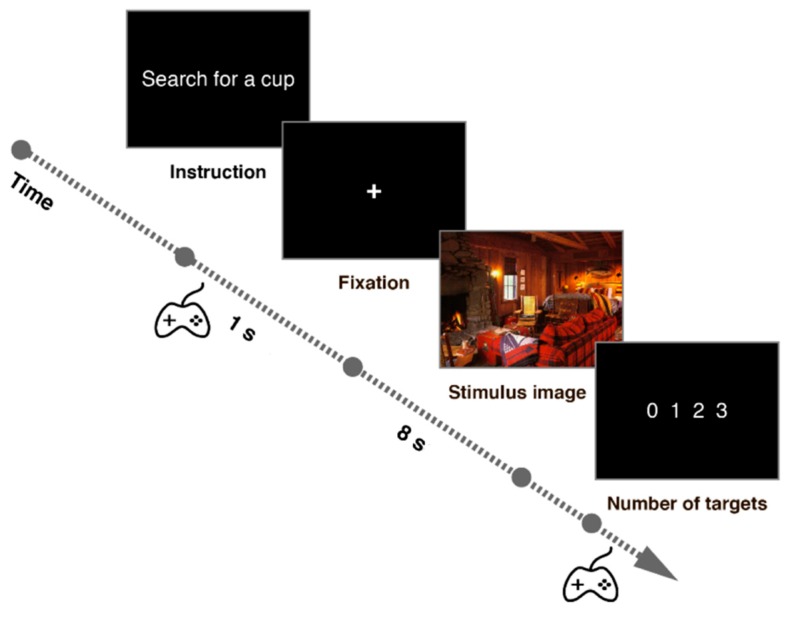
The trial sequence based on the description in [30].

The experiment consisted of two blocks (of 45 and 46 trials), with the goal of searching for “cups” or “paintings”, respectively. The target remained the same within an experimental block, but the order of the blocks and of the images presented within blocks were randomized for each participant. The number of target objects varied for each image, and no participant saw the same image twice. Each trial began with the instruction to search for a particular target shown on the screen until a joystick-button press initiated the trial. A fixation cross was then displayed for one second, until the appearance of the search image. The search image was presented for eight seconds, and participants were asked to search through the image and specify the number of target objects shown after its removal by pressing the joystick up and down buttons. Figure 2 shows the trial sequence. Eye movements were monitored with the Eyelink 1000 eye-tracking system (SR Research Ltd., Ottawa, ON, Canada), sampling at 1000 Hz. Written informed consent was obtained before the experiment from each participant. The experiment and consent form were approved by HSE ethics committee.

The current study used two datasets from the experiment described above. The first dataset contained a subset of the data collected on 44 pictures. Information includes 782 first fixations (29,528 fixations in total). This dataset was used in Experiments 1–3. The second, larger dataset contained data collected on 91 pictures, with 1593 first fixations (60,186 fixations in total), and was used in Experiments 3 and 4. We are providing the total numbers of the observations in the eventual datasets used in the study after all preprocessing and the exclusion of outliers (see below). For our purposes, we extracted the information about the latency of the first fixation made by participants after the onset of the image (i.e., the first fixation where both onset and offset occur while the image is on the screen). Since the WTA layer implements IOR as a separate mechanism from the LIF, we wanted to ensure that we were comparing distributions generated by the LIF layer alone. For this reason, fixations influenced by IOR in both our model, and human data were not tested. In principle, however, latency distributions with and without IOR both have the typical skewed distributions observed in most human responses [32,33]. Eye movement events (such as saccades, fixations, and blinks) were automatically detected by Eyelink algorithms, with saccade detection set at 35 degrees per second [34,35]. Fixations longer than 1500 ms were considered to be outliers and were excluded from further analysis (1% and 3% of data, respectively, to the datasets). The materials are available upon request.

### 2.2. Saliency Model Implementation

**Figure 3:**
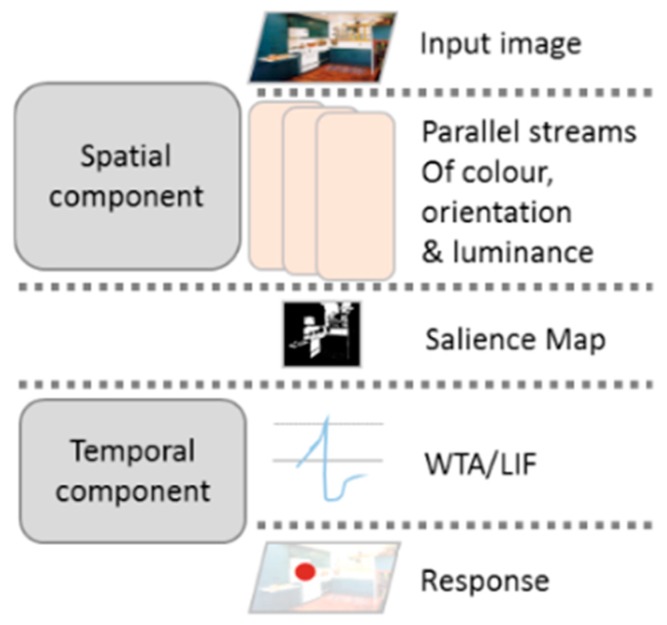
The figure shows the structure of the model used in the study. The spatial component produces a saliency map, which is used as an input for the temporal component, represented by a WTA network. The final outcome of the model is a predicted fixation, with its latency and spatial location.

We modified the algorithm developed by Walther and Koch [5], which is an extended version of the Itti et al. [12] implementation of the Koch and Ullman [3] Saliency Model that accounted for attending to proto-object regions [36] and incorporated feedback connections. The source toolbox code was modified by separating the first-stage saliency map production from the LIF and WTA components of the model’s implementation. Although the method of the model’s prediction was not changed, this allowed testing temporal accuracy and modifying the LIF parameters separately from the spatial saliency map. The final structure of the model is shown in the Figure 3.

In this implementation, salience maps were produced once per image, and fixations in the model were generated by the LIF neurons in the WTA layer based on that salience map. The input current of LIF neurons in the model was initialized to the value of the saliency map for each region, multiplied by a constant for scaling out range, and with some additional added noise. The voltage of each neuron was updated, iteratively, according to the following formula and included all parameters listed in Table 1:

*Vnew* = *Vold* + *dt* · *C* ((*I* − *Gleak* (*V* − *Eleak*) − *Gexc* (*V* − *Eexc*) − *Ginh* (*V* − *Einh*)) (1)

The neuron fires if the membrane potential V is higher than the threshold potential Vthresh. The input conductivity parameter, Ginput, is used to convert the saliency map from the previous layer of the model to the input current, I.

### 2.3. Optimization Procedure

The optimization methods are described here, with details that are common to all experiments. Where the procedures differ, the details will be provided later for each experiment.

The Saliency Model, as provided by the authors, was set with initial parameter values, so we used this as a baseline parameter set whenever initialization was required, as defined in [5].

In order to optimize the parameters in our test versions, we chose to use GA and NM methods. The optimized parameters included the parameters of the LIF component (the potential for excitatory and inhibitory channels, the leak conductivity, the input conductivity, the threshold potential for firing, and capacity) and the WTA component (capacity, the leak conductivity, and the conductivity of inhibitory channels), as well as three noise parameters (the amplitude of random noise, the amplitude of constant noise, and the range of the saliency map output) for all the optimization experiments.

For both optimization algorithms, the fitness function used statistical tests comparing the distribution of human fixation durations to the distribution generated by the model. Specifically, we minimized the statistic values from a combination of statistics from Kolmogorov–Smirnov (KS) test and z-tests of the ground truth (the observed human data) and simulated distributions. Information about particular fitness functions that were used in different runs is provided in Table 2.

The optimization procedure always started with the default parameters suggested in Walther and Koch’s [5] implementation, if not otherwise specified. The search space for the new parameters was not limited. The GA had 40 contenders in each generation, and the mutation range was set up to 10%, with the direction and percentage value randomized on a given instance. The lowest 10 contenders were reset to the initial parameters.

The NM method was used with default stop criteria of the Matlab *fminsearch* function [28,29]. GA was run for 50 generations or until the optimization procedure had found parameters able to produce a distribution statistically indistinguishable from the ground truth by Kolmogorov–Smirnov and Z-test. According to machine-learning practice, larger training datasets lead to better model performance. Based on this fact, we repeated the optimization on the training dataset by using additional data. We increased the human dataset by adding data collected on the other 47 images. There was no separated testing dataset in the study. The final set of parameters for each experiment could be found in Appendix A.

Statistics were obtained by Matlab *ks.test* and *ztest* functions (software version R2014b) during an optimization run and for evaluation of the obtained results. The plots were created in R (version 3.5.2) via ggplot2 package (v 3.2.1); the descriptive statistics were also calculated in R.

## 3. Experiment 1: Testing the Default Parameters

### 3.1. Methods of Experiment 1

First, we tested the temporal predictions of the model with the default parameter space, as defined in [5], against the first saccades taken from the human data in the first dataset.

### 3.2. Results of Experiment 1

**Figure 4:**
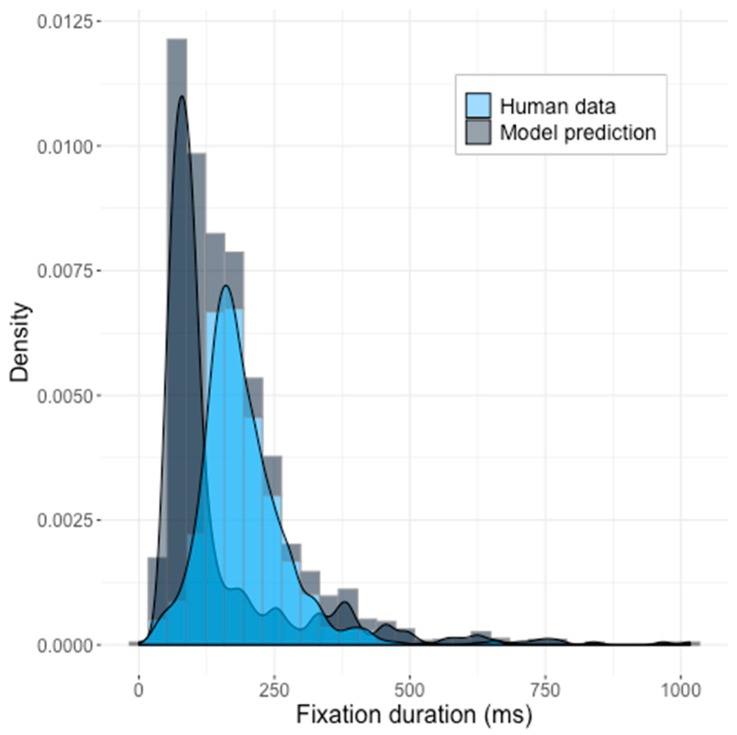
The predictions of the Saliency Model with the default parameters suggested by Walther and Koch [5] against the human data. Z-test statistics: *z* = 16.38, *p* < 2.2 × 10^−16^. KS-statistics *D* = 0.58067, *p* < 2.2 × 10^−16^.

The obtained distribution of predicted reaction times (RT) did not match the observed human data. The histograms of model results and the human data, with the corresponding density functions superimposed, are shown in the Figure 4.

Although the model did produce a skewed distribution, the default model produced shorter fixation durations in comparison to the human data and showed a narrower standard deviation interval (*z* = 16.38, *p* < 2.2 × 10^−16^) (model statistics: *mean* = 150 ms, *SD =* 146 ms; human data: *mean* = 190.8 ms, *SD* = 77.7 ms). We compared the generated distribution of times to those produced by human participants, using the Kolmogorov–Smirnov test, and we were able to dismiss the null hypothesis that the two were sampled from the same distribution (KS-test: *D* = 0.58067, *p* < 2.2 × 10^−16^, see Figure 4).

The default parameter space, therefore, cannot be considered as acceptable for modeling temporal aspects of saccadic movements.

## 4. Experiment 2: Parameter Optimization

### 4.1. Methods of Experiment 2

In order to obtain better temporal accuracy, we optimized the default parameters of the Saliency Model with GA and NM methods. The optimized parameters included all parameters of the LIF component, as well as the three additional noise parameters.

### 4.2. Results of Experiment 2

**Figure 5:**
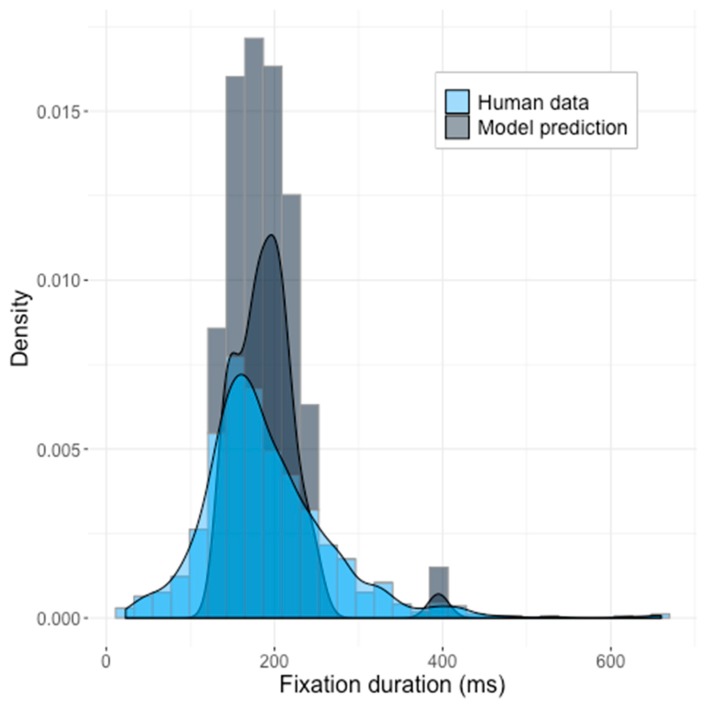
The predictions of the Saliency Model with the optimized set of LIF parameters from Experiment 2 pictured against the human data. The model generated one initial saccade per image (for 44 pictures).

The best result was obtained with the NM method. A z-test showed that the mean and standard deviation of two distributions were not statistically different (*z* = 0.00075273, *p* = 0.9994), however, based on a two-sample Kolmogorov–Smirnov test, we were able to dismiss the hypothesis that model and human data were from the same distribution (*D* = 0.22506, *p* = 0.02939). A visual inspection of the results (Figure 5) also revealed multimodality in the generated data that was not typically observed in human data and likewise did not show typical positive skewness. The Saliency Model showed poor fit to the ground truth.

## 5. Experiment 3: Increased Number of Fixations

### 5.1. Methods of Experiment 3

We next looked into the parameter space to see where possible sources of variability in fixation durations might arise. Considering that the Saliency Model has random components (constant and random noise: parameters noiseAmpl and noiseConst of the Saliency Model) in the LIF layer, there was the possibility that these could improve the fixation-duration distributions. To give this parameter the best chance to influence the resulting distribution, we increased the number of fixations that were generated by the model for each image. Fixations per image were increased to 10, although each of these fixations was generated independently as a ‘first’ fixation (i.e., without IOR).

The experiment consisted of two runs. In the first one, the generated distribution consisting of 440 saccades (10 saccades for 44 images) was compared with the human data. The optimization procedure was the same as in Experiment 2, except for the number of predictions produced by the model. The optimization procedure started with the default parameters in each optimization run.

The larger training datasets could lead to better model performance, so we repeated the optimization on the training dataset by using additional data. We increased the human dataset by adding data collected on the other 47 images; the new dataset had 91 images in total, collected from the same 18 participants. The new dataset contained information about 1593 initial fixations (60,186 fixations totally) (see Section 2.1, “Data Collection”, for more details).

### 5.2. Results of Experiment 3

**Figure 6:**
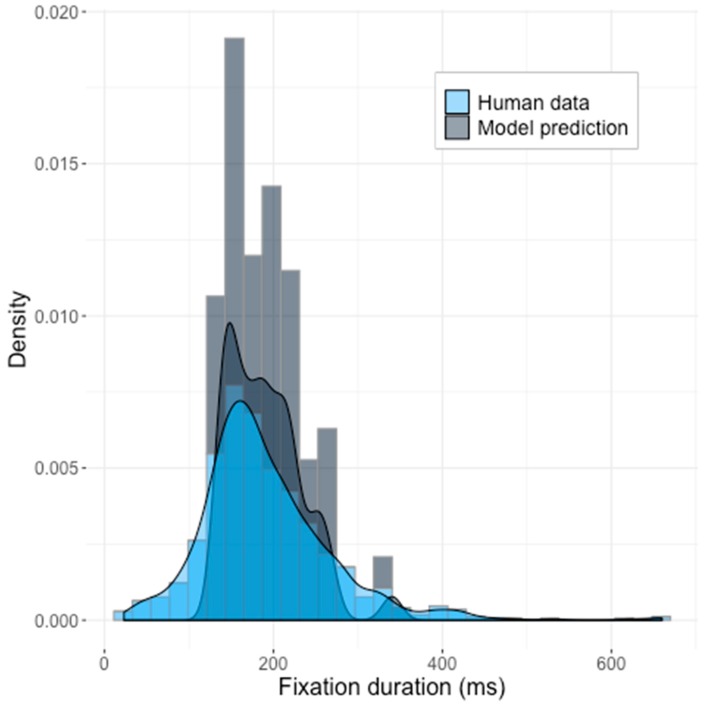
The Saliency Model’s predictions with the third set of parameters shown against the human data. The model generated ten initial saccades per image (for 44 pictures).

The distribution of latencies produced by the best parameters was closer to the ground truth of the human data (Figure 6). The 10 generated saccades per image were compared to the original 44 image dataset, and they matched in means and standard deviations (*z* = –0.013418, *p* = 0.9893), but the KS-test again rejected the null hypothesis that both samples came from the same distribution (*D* = 0.19182, *p* = 2.007 × 10^−9^).

**Figure 7:**
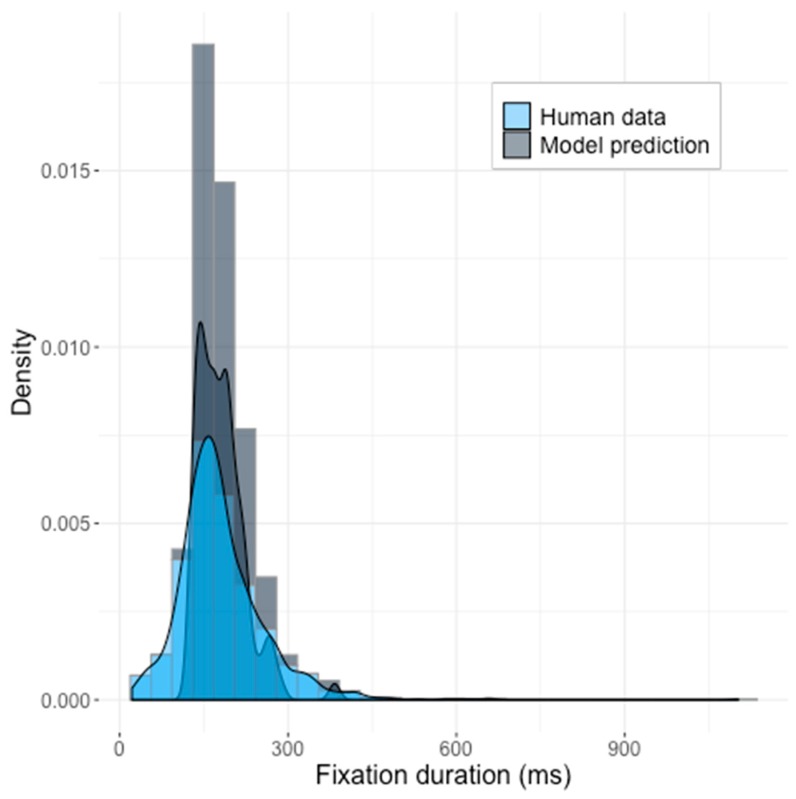
The Saliency Model’s predictions with the fourth parameters space, learned on the 91 pictures’ dataset. The model generated ten initial saccades per image.

For the second optimization run, we used the increased dataset with 91 pictures, resulting in a worse fit with human data (Figure 7) and did not match with the mean of the distribution (*z* = 3.1117, *p* = 0.00186), the KS-test also showed unsatisfying of the results (*D* = 0.15857, *p* = 1.305 × 10^−9^).

The analysis revealed that noise, which was supposed to increase similarity to the real human data, did not produce enough randomness in the model predictions. The default amplitude of random noise was 10^−17^, and the amplitude of constant noise was 10^−14^. This was apparently too small, and the model seemed to learn to produce a distribution of the fixation durations based only on differences in saliency of different images. This, of course, differed from the ground truth of human reactions.

## 6. Experiment 4: Learning New Parameters on Data Separated by Pictures

### 6.1. Methods of Experiment 4

**Figure 8:**
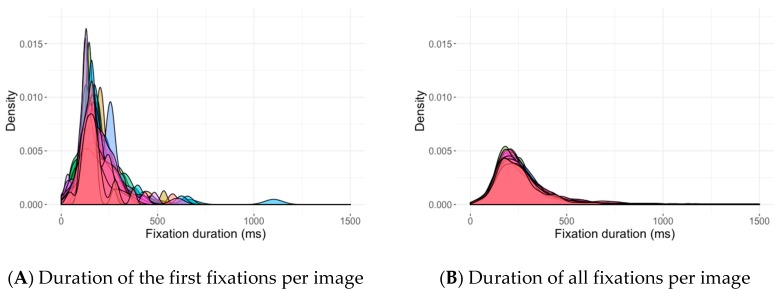
The distributions of saccades’ latency for each image in the dataset. On the left: only initial saccades (16–19 data points for each image). On the right: all saccades (542–794 data points for each image, mean number 661). Both plots show that latencies of saccades, which are related to only one image, nonetheless tend to produce a right-skewed distribution, typical for any RT distribution.

Our human dataset contained 16–19 initial fixations per image (pooled from all participants), and, as expected, there was natural variability in duration of initial fixations for the same image across participants (see Figure 8). The shape of the fixation duration distribution again has typical positive skewness of RT distribution, even for the data obtained on one image (Figure 8). Since the model did not learn to use noise to produce various outputs, we restricted it to determine whether it could predict the duration of initial fixations given a particular picture.

The next version of the training algorithm was used to prevent our model from relying on different levels of input saliency to produce the distribution. The human data were divided into images, and an optimization function was set up as a sum of KS-statistics calculated for model predictions against the ground truth for each image separately. Since we only had a limited number of first human fixations per image, we used data from all fixations as the ground truth, to train the parameters for the image distributions.

### 6.2. Results of Experiment 4

**Figure 9:**
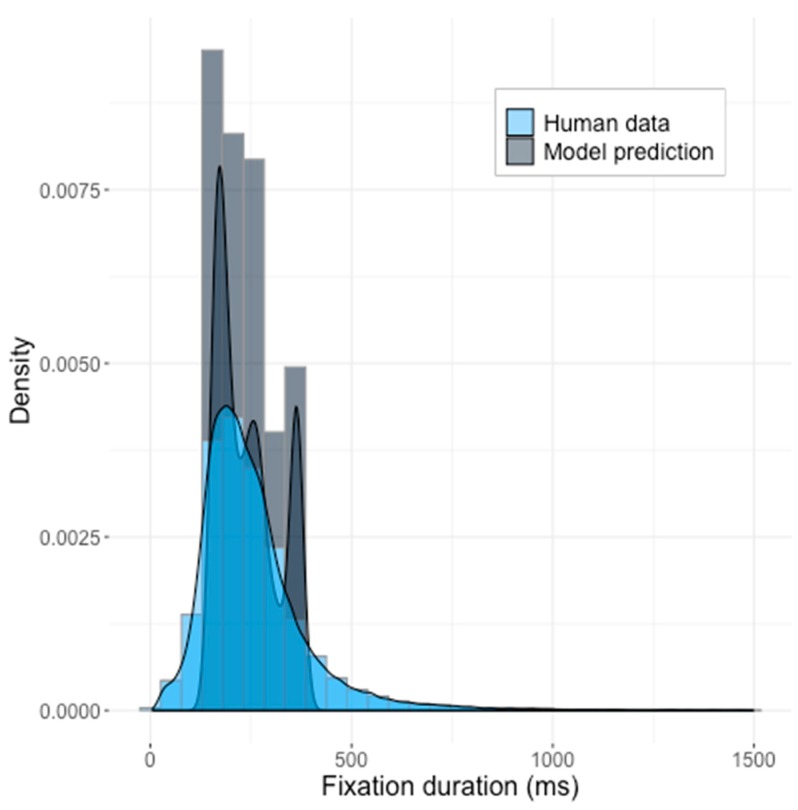
Saliency Model’s predictions with the fifth parameters space, learned on the 91 pictures dataset. The model generated ten initial saccades per image. Z-test statistics: *z* = 6.5611, *p* = 5.341 × 10^−11^; KS-statistics: *D* = 0.19327, *p* < 2.2 × 10^−16^.

The best parameters of the trained model confirmed our concerns showing that the algorithm was not able to find an accurate set of parameters when forced to incorporate variance from its own parameters rather than from the images. The distribution produced by the resulting parameter space was not close to the ground truth of human data (*D* = 0.19327, *p* < 2.2 × 10^−16^; see Figure 9).

**Figure 10:**
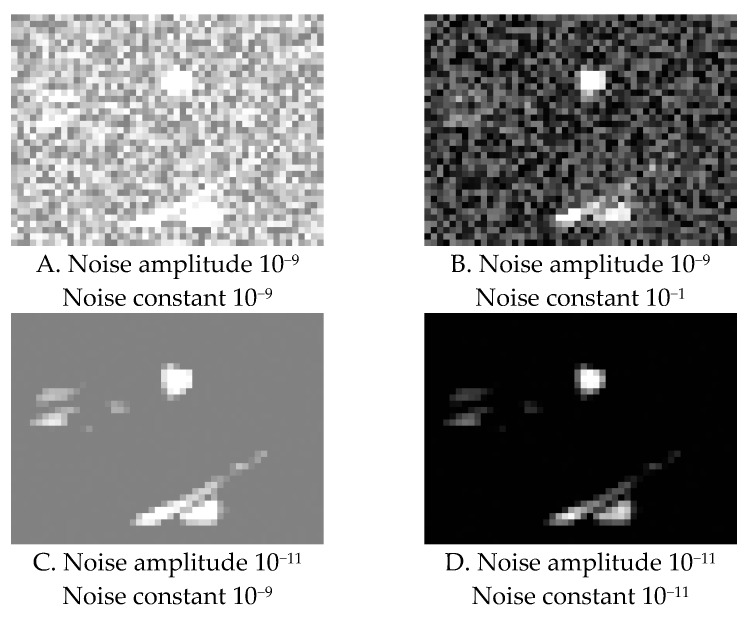
Examples of an effect of different noise parameters on the saliency map. Adding more noise only changes the saliency map, which is used as initial data, but does not modify the integration and firing process itself.

The best noise constants were 10^−14^ and 10^−11^ for random and constant noise, respectively, and were able to produce a maximum of two different fixation duration values per image. An attempt to increase the parameters manually resulted in a model that failed to generate a saccade within a two-second interval. On the other hand, the version with manually decreased parameter values was able to produce only one fixation response per image. These results are due to the fact that these parameters add the noise directly to the saliency map, which typically has features of the order 10^−9^, as shown on Figure 10. The noise does not change the fact that the most salient location triggers the spike and that particular neuronal spike is deterministic and defines the time of a gaze shift: therefore, the noise in this model does not impact the dynamics of the model as such but changes its initial values, which explains low variability between different runs on the same image.

## 7. Discussion

We tested the temporal accuracy of the classic LIF + WTA salience model architecture in order to determine whether any parameter space of the model could provide accurate temporal fitting of observed human data.

The default parameters did not reproduce the temporal dynamics of human visual attention, nor did the model with multiple attempts to find optimized parameters. Further investigation showed that the result was highly dependent on variability in the input images, with additional images even making the final distribution worse. The noise parameters in LIF do not bring much change to the fixation durations, and they turn them into constant values rather than into samples from a continuous distribution.

The classic LIF approach is a biologically plausible model of neural-level accumulation that theoretically could predict both spatial and temporal aspects of fixations and saccadic eye movements. The great advantage of the LIF neuron is that this model could be considered a connecting link between low-level modeling of neural activity and modeling of high-level cognitive processes. LIF was shown to be an accurate model of a single-neuron spiking behavior (and the modified version, generalized leaky integrate-and-fire, GLIF, can accommodate different types of neurons [37]). Moreover, LIF units combining in a spiking network were shown to successfully perform demanding cognitive tasks (such as image processing, solving arithmetic problems, etc. [38]), with a similar performance level to classical deep artificial neural networks (see [39] for the review).

However, taken with its default parameters, LIF has been shown to be limited in simulating temporal behavior accurately. Our best results did find a parameter fit that was not different from human data in terms of mean and standard deviation and, to a lesser degree, the overall distributions. The latter, however, was only achieved by using the variance between images, to generate a realistic distribution, and is a crucial test if we want to evaluate the validity of the assumptions taken by different models [21]. Other demonstrations [32,33] have shown that the difference between a bias shift and true improvement in an attentional task cannot be seen in mean RTs, but only in the change in distribution shape.

This result will probably hold true for any implementation of a biological neuron model that does not include an intrinsic source of noise during the integration part. As neuron models more complex than LIF still usually do not provide a source of intrinsic noise, those models should not perform well at approximating the RT distribution as well.

Other models have been shown to simulate visual search with a high degree of accuracy. For example, guided search uses an early race component to model competition between early features, but this is a small component in a larger model. Drift diffusion models perform extremely well on response distributions [24,32]; however, they restrict the problem by allowing only two signals to ‘race’ toward a single threshold. Finally, there are combinations of classic diffusion algorithms and LIF, the so-called ‘leaky competing accumulator’ models [23], which include biologically important features, e.g., an accumulation drop during signal loss (leaky) and lateral inhibition. However, with this implementation, signals are spatially abstracted and inhibit all other signals, whereas, in the classic LIF model, a neuron inhibits only its adjacent neurons.

Despite showing high levels of temporal accuracy, leaky competing accumulator models are similar to other accumulation models in that they lack a true spatial component. The LIF algorithm represents a true 2D map of visual space, whereas accumulator models abstract space into a number of key locations, without consideration of their actual proximity. The saliency map may be used to influence the parameter choice in these accumulator models [40,41], but the accumulator is not a model of the retinal salience map per se. Accumulators are good at modeling experimental results obtained under laboratory conditions [25] but they are not applicable to natural scene processing without first abstracting key locations.

A possible way to overcome current limitation and improve accuracy and comprehensiveness of the model would be the combination of saliency (as a foundation for spatial predictions) and a race model, such us drift diffusion model or leaky competing accumulator model, (for temporal prediction) for better results.

Another possible future direction of the research could be an investigation of temporal dynamics of the Target Acquisition Model (TAM), proposed by Zelinsky [42,43]. This model provides an alternate account of human perception, and, like the Itti and Koch’s model, it is well-grounded in cognitive and neurobiological theory. TAM is able to model gaze-shift latency, but the noise component is also added to the target map generation stage. Zelinsky and co-authors have considered the problem of temporal dynamics and reported that their model could suffer from the same problem; however, the model’s ability to predict eye movement latencies has not been fully tested [44].

To sum up, no current model of visual search generates an accurate model of the full response-time distributions and spatial locations of saccades. The classic Saliency + LIF model produces both spatial and temporal data, but it does not output an accurate temporal distribution of RTs, as it does not have a source of randomness needed to output a distribution.

## Figures and Tables

**Table 1:** LIF parameters of the Saliency Model [5]. For more details about the specific implementation, see [18] and the Matlab SaliencyToolbox [26].

Parameter	Meaning
dt	time step for integration (1 × 10^−3^ ms)
Eleak	leak potential
Eexc	potential for excitatory inputs
Einh	potential for inhibitory inputs
Gleak	leak conductivity
Gexc	conductivity of excitatory channels
Ginh	conductivity of inhibitory channels
GinhDecay	time constant for decay of inhibitory conductivity
Ginput	input conductivity
Vthresh	threshold potential for firing
C	membrane capacitance
V	current membrane potential
I	current input current

**Table 2:** Summary of the optimization procedure parameters used in the study.

Optimization Procedure	Generated Data Sample Size	Ground Truth Data
	Experiment 1	
No optimization (the default parameters were used)	44 data points	44 images first (initial) fixations duration only sample size *N* = 782
	Experiment 2	
Optimization functions that were used in different runs - for GA (1) Kolmogorov–Smirnov statistic + Z-test statistic (2) First the contenders that didn’t pass Z-test were sorted to the end of the list; than the rest were compared by Kolmogorov–Smirnov statistic - for NM method (1) Kolmogorov–Smirnov statistic + Z-test statistic	44 data points	The same as in Experiment 1 (44 images first fixations duration only sample size *N* = 782)
	Experiment 3	
Optimization functions were the same as in Experiment 2	(1) 440 data points (2) 910 data points	(1) The same as in Experiment 1 (44 images first fixations duration only sample size *N* = 782) (2) 91 images the first fixations duration only sample size *N* = 782
	Experiment 4	
- for GA: sum of Kolmogorov–Smirnov statistic obtained on each image. - for MN-method: sum of Kolmogorov–Smirnov statistic obtained on each image	910 (10 per image)	91 images mean sample size per image n = 661, total *N* = 60,186 (all fixations, not only the first ones)

## Appendix A

The final parameters in each optimization experiment are provided below.

### Appendix A1. Experiment 2.

wta.sm

    Eleak: 0,
    Eexc: 0.1065906219,
    Einh: −0.02107685958,
    Gleak: 9.894609018e-09,
    Gexc: 0,
    Ginput: 5.337295999e-08,
    Vthresh: 0.0008468968888,
    C: 1.102496522e-09,

wta.exc

    Eleak: 0,
    Eexc: 0.1041268439,
    Einh: −0.02068941848,
    Gleak: 9.356995909e-09,
    Gexc: 0,
    Ginh: 0,
    Ginput: 2.656232694e-08,
    Vthresh: 0.001210968391,
    C: 1.03337653e-09,

wta.inhib

    Eleak: 0,
    Eexc: 0.1030162526,
    Einh: −0.02116698772,
    Gleak: 9.596446818e-09,
    Gexc: 0,
    Ginh: 0,
    Ginput: 5.105792852e-08,
    Vthresh: 0.001018027772,
    C: 1.020098518e-09,

timeStep: 0.001,

smOutputRange: 1.108206351e-09,

noiseAmpl: 9.942605797e-16,

noiseConst: 8.879142166e-13.

### Appendix A2. Experiment 3

(1) The first run

wta.sm

    Eleak: 0,
    Eexc: 0.1009250677,
    Einh: −0.02007224066,
    Gleak: 1.02699624e-08,
    Gexc: 0,
    Ginput: 4.983690833e-08,
    Vthresh: 0.0005360834937,
    C: 1.076267842e-09,

wta.exc

    Eleak: 0,
    Eexc: 0.09885402437,
    Einh: −0.01942183543,
    Gleak: 1.03233152e-08,
    Gexc: 0,
    Ginh: 0,
    GinhDecay: 1,
    Ginput: 4.405453667e-08,
    Vthresh: 0.001140167888,
    C: 1.109382579e-09,

wta.inhib

    Eleak: 0,
    Eexc: 0.09633707116,
    Einh: −0.02326386049,
    Gleak: 1.008793641e-08,
    Gexc: 0,
    Ginh: 0,
    Ginput: 5.04747311e-08,
    Vthresh: 0.001018984672,
    C: 1.026927422e-09,

timeStep: 0.001,

smOutputRange: 1.062391456e-09,

noiseAmpl: 1.018242784e-15,

noiseConst: 9.88642386e-13.

(2) The second run

wta. sm

    timeStep: 0.001,
    Eleak: 0,
    Eexc: 0.1052790164,
    Einh: −0.02132743015,
    Gleak: 9.792812288e-09,
    Gexc: 0,
    Ginput: 5.278942991e-08,
    Vthresh: 0.0005497130693,
    C: 8.94918705e-10,

wta.exc

    timeStep: 0.001,
    Eleak: 0,
    Eexc: 0.1045285492,
    Einh: −0.02129696363,
    Gleak: 1.127142927e-08,
    Gexc: 0,
    Ginh: 0,
    Ginput: 4.216897711e-08,
    Vthresh: 0.001043880035,
    C: 1.158786096e-09,

wta.inhib

    timeStep: 0.001,
    Eleak: 0,
    Eexc: 0.1042935178,
    Einh: −0.02066701205,
    Gleak: 1.05123827e-08,
    Gexc: 0,
    Ginh: 0,
    Ginput: 4.704205093e-08,
    Vthresh: 0.001004900021,
    C: 1.049336853e-09,

timeStep: 0.001,

smOutputRange: 9.210187215e-10,

noiseAmpl: 1.016741992e-15,

noiseConst: 1.010360845e-12.

### Appendix A3. Experiment 4

wta.sm

    Eleak: 0,
    Eexc: 0.1053274615,
    Einh: −0.01834220167,
    Gleak: 1.118348755e-08,
    Gexc: 0,
    Ginput: 4.831627415e-08,
    Vthresh: 0.001091101504,
    C: 1.048042556e-09,

wta.exc

    Eleak: 0,
    Eexc: 0.1013037294,
    Einh: −0.02034546987,
    Gleak: 1.136629615e-08,
    Gexc: 0,
    Ginh: 0,
    Ginput: 1.820687988e-08,
    Vthresh: 0.001192885343,
    C: 8.696685367e-10,

wta.inhib

    Eleak: 0,
    Eexc: 0.1005701907,
    Einh: −0.02148144476,
    Gleak: 9.585624857e-09,
    Gexc: 0,
    Ginh: 0,
    Ginput: 5.459659917e-08,
    Vthresh: 0.001027481599,
    C: 9.502088856e-10,

timeStep: 0.001,

smOutputRange: 1.163724762e-09,

noiseAmpl: 9.696348623e-15,

noiseConst: 8.965883592e-12
